# Correction: Internet of Things (IoT) for Smart Agriculture: Assembling and assessment of a low-cost IoT system for polytunnels

**DOI:** 10.1371/journal.pone.0296110

**Published:** 2023-12-14

**Authors:** Nuwan Jaliyagoda, Sandali Lokuge, P. M. P. C. Gunathilake, K. S. P. Amaratunga, W. A. P. Weerakkody, Pradeepa C. G. Bandaranayake, Asitha U. Bandaranayake

There are errors in the Funding statement. The correct Funding statement is as follows: This work was carried out with the aid of a grant from UNESCO and the International Development Research Centre, Ottawa, Canada. The views expressed herein do not necessarily represent those of UNESCO, IDRC or its Board of Governors.
