# Supplementary figures and images for: Internet of things (IoT) for smart agriculture: Assembling and assessment of a low-cost IoT system for polytunnels
Source: PLoS One. 2023 May 25;18(5):e0278440. doi: 10.1371/journal.pone.0278440 (PMC10212138; doi:10.1371/journal.pone.0278440)

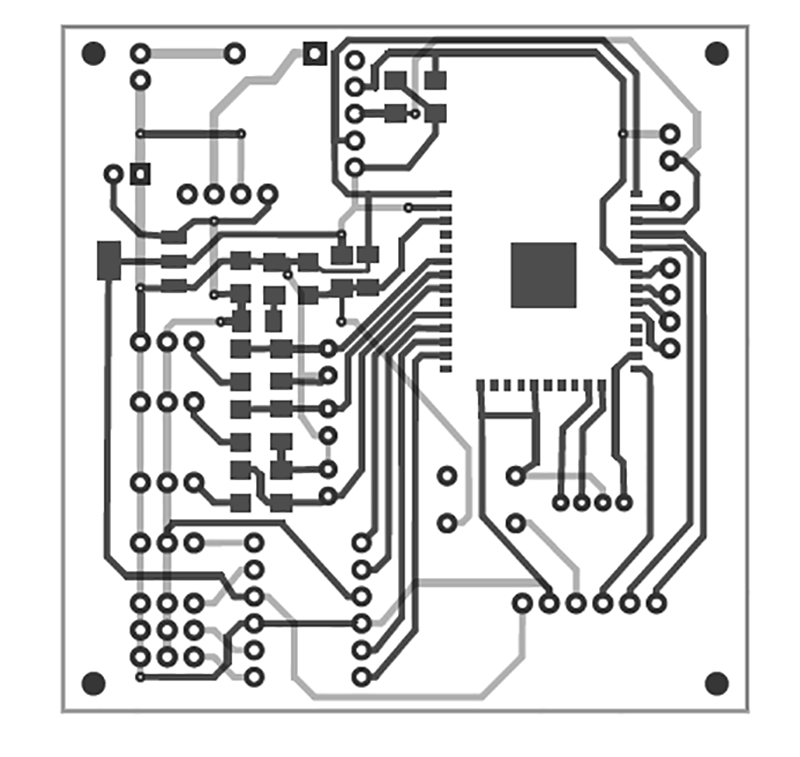

Supplement: S1 Fig — ESP32 was used as the microcontroller of the sensor node, since it is a low-cost, low-power SoC (System on Chip), with a dual-core Tensilica Xtensa LX6 microprocessor that works at 160MHz. It is specially designed for wireless IoT sensor applications with necessary modules for GPIO as well as in-built WiFi and Bluetooth functionalities. (TIF) [file pone.0278440.s001.tif]

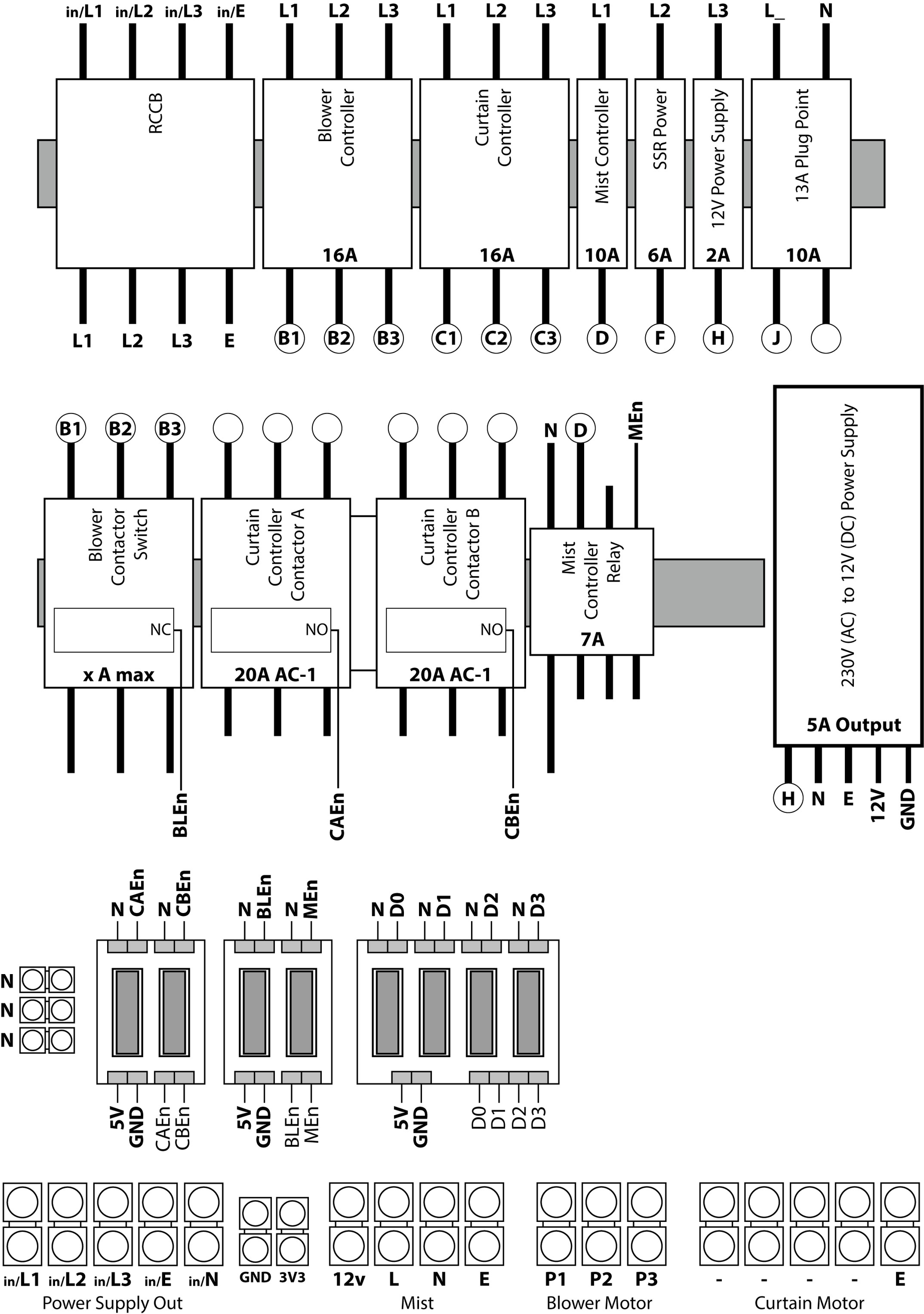

Supplement: S2 Fig — High level panel design of the controller, which handles the signal given by ESP32 microcontroller (SoC). There are 8 digital outputs for controlling the devices connected to the controller panel. BLEn, MEn, CAEn, and CBEn are the main output signals and D0-D3 are auxiliary output control signals from the microcontroller. There are 2 optically isolated inputs in the controller unit, to obtain the limit signals from the curtain controller. One input to get the curtain to reach its down-limit signal and one input to get the curtain reached to its up-limit signal. (However, the curtain controller isn’t used as a control parameter in this study) Four status indicators were used to indicate the status of the controller node, a power indicator, a WiFi connectivity indicator, a control signal indicator, and an error condition indicator. (TIF) [file pone.0278440.s002.tif]
