# Supplementary material for: Internet of things (IoT) for smart agriculture: Assembling and assessment of a low-cost IoT system for polytunnels
Source: PLoS One. 2023 May 25;18(5):e0278440. doi: 10.1371/journal.pone.0278440 (PMC10212138; doi:10.1371/journal.pone.0278440)
Supplement: Attachment — Submitted filename: Rebuttal Letter.pdf [file pone.0278440.s003.pdf]

General Comments

In your Methods section, please provide additional information regarding the permits you obtained for the work. Please ensure you have included the full name of the authority that approved the field site access and, if no permits were required, a brief statement explaining why.

> According to the current rules and regulations in the country, no specific permission or approvals were required from any organization to conduct this research. (Mentioned in Page 5)

Please note that PLOS ONE has specific guidelines on code sharing for submissions in which author-generated code underpins the findings in the manuscript. In these cases, all author-generated code must be made available without restrictions upon publication of the work. Please review our guidelines at https://journals.plos.org/plosone/s/materials-and-software-sharing#loc-sharing-code and ensure that your code is shared in a way that follows best practice and facilitates reproducibility and reuse.

> We are also happy to make our works open-source. A part of the technical information comes under the *support information* document in the submission. The rest of the implementation details with source-code is openly available in the following code repository, https://github.com/cepdnaclk/Smart-Agriculture-a-low-cost-IoT-system-for-polytunnels

In your Data Availability statement, you have not specified where the minimal data set underlying the results described in your manuscript can be found. PLOS defines a study's minimal data set as the underlying data used to reach the conclusions drawn in the manuscript and any additional data required to replicate the reported study findings in their entirety.

> We published our datasets to the public and the access details to the datasets mentioned on the Page 21

Reviewer #1

The manuscript has the scholarly importance and it seems look very interesting and to appreciate the authors for their scientific efforts, but need to improve the article is presented in an intelligible fashion and thoroughly check most of word combined (e.g.: polytunnels, Testbed etc) and not fit as per POLS guidelines. These are the following suggestion and comments for author to incorporate before final publication.

> We go through your review comment, and update the term ‘Testbed’ with ‘Test bed’ (Page 14). However, ‘polytunnel’ is considered a single word in most of the places in the literature, so we kept it as it is.

1. There is no specific logic mentioned for selecting the area standard of poly-tunnel, in order to place sensor and control the metrological parameters as mentioned under heading Sensor Node design.

> As per your comment, we further explained the design designs on both the sensor node and controllers node under the section “System design” (Page 7-9)

2. Under heading communication it is not represents clear meaning “Since both greenhouses were located nearby, no Wi-Fi repeaters were required”. Need to clearify.

> We have addressed that part and included more generalized reasoning for the above statement in the Page 11

3. Conclusion should be

4. Please revise your conclusion part separately into more details. Basically, you should enhance your contributions, limitations, underscore the scientific value added of your paper, and/or the applicability of your findings/results and future study in this session.

> The conclusion section was revised as suggested (Page 24-26).

<u>Reviewer #2</u>

1. Numbering specific objectives in the abstract should be removed

> Revised as suggested (Page 1).

2. The research papers demonstrates originality to develop and test IoT system for achieving sustainable goals in agriculture practice.

3. The work is logically designed to develop IoT using basic blocks. However, the word low-cost is not clearly justified... If there is already, an existing IoT system used to monitor greenhouse plants using control parameters like temperature, humidity, etc. I wish the authors could add a section on the IoT cost for smart agriculture and how this system is different.

> Since it is a little difficult to directly include the prices with references since it is subjective to change from time to time as well as not possible according to the journal policies.
>
> However, a new section was added under, “Materials and Methods”, as “Cost Estimations”, where we discuss the cost-related content and justifications, especially the setup cost and the operational cost (Page 12-14).

4. Certain IoT functions like monitoring, controlling are addressed.

5. The polytunnel experiment results showed a reduction of 3.6 oC temperature. What is the change in temperature and humidity for a 24-hr period in the polytunnel without use of sensors? In the control polytunnel, you are using blowers and misters for controlling the temperature. How does this step go to prove the statement of developing a low-cost IoT. These are power-driven devices, which add to the cost of managing the polytunnel.

> There is no polytunnel without sensors. Both polytunnels consist of sensors while one of them also has a control system.
>
> In this study, our primary target was to reduce the cost of setup. The operational cost mainly depends on the control devices, in this case, the blowers and misters. We didn’t compare the operational cost of the system, but it is a good suggestion to take consider in the next phase of this research.

6. The effect of changes in environment conditions of polytunnel and plant yield should be highlighted.

> Several crop cycles are completed so far and there is a comprehensive dataset was collected on crop growth, physiology, gene expression, agronomy, etc. The newly developed system presented in this manuscript was utilized for the experiments. The objective was to show the adaptation of the selected variety (tomato) to climate change. Therefore, the data set and analysis do not match the theme of this manuscript. That is the reason to only present the system and its performance in this manuscript.

Reviewer #3

The paper entitled “Internet of Things (IoT) for Smart Agriculture: Assembling and assessment of a low-cost IoT system”, although is utterly interesting and addresses to the readership of the Journal, appears to have some issues in which the authors should put more effort:

1. The authors should reconsider the title of their manuscript taking into account the fact that it mostly refers to controlled agricultural environments such as greenhouses and not open arable areas.

> We also agree with the suggestion, and therefore we have updated the title of our study to, “Internet of Things (IoT) for Smart Agriculture: Assembling and assessment of a low-cost IoT system for polytunnels”.

2. The subject of research is not entirely new, since several works have been published on it. Although the authors cite a relatively high number of references they fail to substantiate the novelty as well as the scientific contribution of their work. To this end, the authors should rework the content of the introduction section by enriching it with a number of references targeting on the subject of:

(a) the current trends and challenges in the Deployment of IoT Technologies for climate smart facility agriculture as for instance the work in: https://doi.org/10.1504/IJSAMI.2019.101673

(b) the IoT technologies in smart greenhouses such as for instance the work in: https://doi.org/10.1016/j.compag.2022.106993.

Finally, additional justifications should be included in the final paragraph of the introduction

> We have updated the content to reflect the objective we had, to work on designing a low-cost and low-power, IoT-based network of devices, which can monitor the environment inside a polytunnel with 3-dimensional coverage on temperature and humidity variations, as well as 2-dimensional coverage of the light intensity received. The idea on 3-dimensional and 2-dimensional measurement coverage of polytunnels is not found yet in the literature as per our knowledge.
>
> Further, we compared the result we obtained with several different environment setups and represented in graphically, which will be helpful for researches who are willing to go further in the research domain.

3. The methodology which is followed in this work as well as the deriving results are not clearly defined. To be more precise, the system design as well as the results seem to be rather superficial whereas the lack of Figures in the entire manuscript does not assist the work of this reviewer in comprehending critical points of the methodology as the system architecture for instance. As for the results, the authors include a number of tables with data regarding temperature, humidity, etc., they do not however present tables with data regarding the performance of the system such as response time, number of requests, energy consumption, etc. This issue has to be attended.

> As mentioned in the introduction section, the main research gap in this domain is not having sufficient real data to come up with innovative solutions. As a solution to that, we decided to share the experience we obtained in designing a system that can collect the required data, with all the design decisions that may be useful to continue. The results section was dedicated to showing the findings of our experiments with the observations we had.
>
> When come to performance, the system we developed is not a hard real-time system, so the response time and the performance of the system based on a number of requests haven’t a significant role. Those topics were already studied under the domain of IoT, and we followed the industry standards developed according to the outcomes of those studies while designing the system.

4. The conclusion section is rather short and generic. The findings of the research and their implications should be discussed in the broadest context possible while limitations on the subject of research should be also further highlighted. A discussion section would aid in this direction.

We improved the Discussion and Conclusion sections as suggested (Page 22-26)

5. The paper is written in good English however some spell and grammar checking is required.

We have revised the manuscript as suggested.

* Please note that the page numbers mentioned here are from the revised manuscript, without tracked changes.
